# Resistance to thyroid hormone alpha associated with a novel THRA ligand-binding domain variant: familial clinical, biochemical, skeletal, and exploratory cerebral FDG-PET/CT metabolic findings

**DOI:** 10.3389/fendo.2026.1891019

**Published:** 2026-10-12

**Authors:** Denisse Mata-Salgado, Juan José Morales-Suárez, Alfredo Adolfo Reza-Albarrán, Rodrigo Hernández-Ramírez, Miguel Angel Corza-Ayala, José Antonio Cienfuegos-Alvear, Ana María González-Jaimes, Iván Martínez-Duncker, Carmen Alaez-Verson

**Affiliations:** 1 Laboratorio de Glicobiología Humana y Diagnóstico Molecular, Centro de Investigación en Dinámica Celular, Instituto de Investigación en Ciencias Básicas y Aplicadas, Universidad Autónoma del Estado de Morelos, Cuernavaca, Mexico; 2 Departamento de Genética, Instituto Nacional de Ciencias Médicas y Nutrición Salvador Zubirán, Ciudad de Mexico, Mexico; 3 Departamento de Endocrinología, Instituto Nacional de Ciencias Médicas y Nutrición Salvador Zubirán., Ciudad de Mexico, Mexico; 4 Departamento de Medicina Nuclear, Instituto Nacional de Ciencias Médicas y Nutrición Salvador Zubirán, Ciudad de Mexico, Mexico; 5 Departamento de Radiología e Imagen, Instituto Nacional de Ciencias Médicas y Nutrición Salvador Zubirán, Ciudad de Mexico, Mexico; 6 Laboratorio de Diagnóstico Genómico, Instituto Nacional de Medicina Genómica, Ciudad de Mexico, Mexico

**Keywords:** FT3/FT4 ratio, resistance to thyroid hormone alpha (RTHα), reverse triiodothyronine (rT3), THRA gene, TRα (thyroid hormone receptor alpha)

## Abstract

**Background:**

Thyroid hormones (TH) regulate skeletal development, central nervous system maturation, and energy homeostasis, primarily through the nuclear receptors TRα and TRβ, encoded by the *THRA* and *THRB* genes. Pathogenic variants in *THRA* lead to resistance to thyroid hormone alpha (RTHα), a rare and diagnostically challenging condition.

**Methods:**

A 36-year-old woman and her 12-year-old son were studied to characterize the clinical, biochemical, radiological, and exploratory cerebral metabolic findings associated with a previously unreported, likely pathogenic variant in *THRA*. Both individuals underwent a comprehensive clinical evaluation. Thyroid function tests included TSH, fT4, fT3, TT3, TT4, and rT3, along with additional biochemical assessments.

Molecular analysis included whole-exome sequencing in proband 1, which identified a variant in *THRA* (NM_199334.5:c.662T>C; p.Ile221Thr), subsequently confirmed in the second case. Additionally, the adult patient underwent brain positron emission tomography with 18F-fluorodeoxyglucose (18F-FDG PET) to assess regional cerebral metabolism with exploratory visual and ROI-based comparison with an age- and sex-matched control subject.

**Results:**

Both patients exhibited a phenotype consistent with RTHα, characterized by hypotonia, macrocephaly, dysmorphic features, chronic constipation, and normocytic anemia. The fT3/fT4 ratio was elevated (high-normal), and rT3 levels were decreased in both cases. The *THRA* variant was located in the ligand-binding domain and segregated with the familial phenotype. Exploratory brain ^18F-FDG PET assessment in Proband 1 showed symmetrically higher FDG uptake in the prefrontal cortex, basal ganglia, and lateral temporal cortex compared with the control subject, with consistently higher SUVmax values across the evaluated ROIs. These findings represent an exploratory single-subject comparison and have not previously been reported in RTHα.

**Conclusions:**

This study expands the clinical, biochemical, radiological, and metabolic spectrum associated with *THRA* variants and suggests that cerebral metabolic evaluation may provide additional diagnostic insights in RTHα. Further studies, including larger cohorts and functional analyses, will be necessary to confirm the proposed mechanisms and the clinical relevance of the observed metabolic findings.

## 1 Introduction

Thyroid hormones (TH) play an essential role in skeletal development and growth, central nervous system (CNS) maturation, cardiovascular and gastrointestinal function, and the maintenance of energy homeostasis. Their synthesis and secretion are regulated by the hypothalamic-pituitary-thyroid axis through negative feedback mechanisms, ensuring adequate hormone levels according to the specific requirements of each tissue (1, 2). Thyroxine (T4), the main hormone secreted by the thyroid gland, acts predominantly as a prohormone that is converted in peripheral tissues into triiodothyronine (T3), the principal physiological ligand of thyroid hormone receptors (TRs), or into reverse triiodothyronine (rT3), which is considered inactive at the nuclear level, although it may exert rapid effects through non-genomic mechanisms (1, 3–6).

TRs are the main mediators of the genomic actions of TH. These nuclear receptors contain a DNA-binding domain and a C-terminal ligand-binding domain (LBD). Upon T3 binding, TRs undergo conformational changes that promote corepressor release and coactivator recruitment, thereby regulating the transcription of target genes (7, 8).

TRs are encoded by *THRA* (Gene ID: 7067) and *THRB* (Gene ID: 7068), each of which generates multiple isoforms through alternative promoter usage and alternative splicing. However, only the TRα1, TRβ1, and TRβ2 isoforms are capable of binding T3 with functional relevance, although their tissue distribution differs. TRα1, encoded by *THRA*, is widely expressed, with prominent expression in the heart, intestine, bone, adipose tissue, and skeletal muscle. In contrast, TRβ1 and TRβ2, encoded by *THRB*, show distinct expression patterns, with TRβ1 predominantly expressed in the liver and TRβ2 mainly expressed in the hypothalamus, pituitary, retina, and cochlea (7, 9, 10).

Pathogenic or likely pathogenic variants in THRA or THRB cause resistance to thyroid hormone alpha (RTHα) or beta (RTHβ), respectively. RTHβ is estimated to occur in approximately 1 in 40,000 live births, whereas RTHα is an extremely rare disorder with an unknown population frequency, with fewer than 50 cases reported to date (11–13).

The diagnosis of RTHα (OMIM #614450) is particularly challenging because conventional thyroid function tests may be normal or show only subtle abnormalities. The most characteristic biochemical pattern includes normal thyroid-stimulating hormone (TSH) levels, low-normal or subnormal total and free thyroxine (TT4 and fT4) levels, high-normal or elevated total and free triiodothyronine (TT3 and fT3) levels, and low rT3 levels; however, this pattern is not consistently observed, making timely diagnosis challenging (1, 4, 14–29).

The fT3/fT4 ratio may represent a useful parameter; however, its diagnostic utility is limited. This limitation is explained by the phenotypic heterogeneity observed among patients with *THRA* variants, variability in genotype–phenotype correlations, and inconsistent biochemical abnormalities across reported cases. Collectively, these factors hinder the standardization of this index as a reliable diagnostic marker; therefore, its interpretation should always be performed within an integrated clinical and molecular context (1, 4, 15, 16, 18, 20, 23, 26, 27).

To further characterize the phenotypic spectrum of RTHα and expand the molecular landscape of *THRA* variants, we evaluated two individuals from the same family through comprehensive clinical, biochemical, and molecular assessment. Whole-exome sequencing initially identified in Proband 1 a previously unreported likely pathogenic variant in the *THRA* gene (NM_199334.5:c.662T>C; p.Ile221Thr), which was subsequently confirmed in his 12-year-old son (Proband 2). Both individuals presented with clinical manifestations consistent with RTHα.

In addition, Proband 1 underwent ^18^F-FDG positron emission tomography (PET) and skeletal radiographic evaluation. PET revealed a symmetric pattern of cerebral hypermetabolism, a pattern that, to our knowledge, has not previously been reported in RTHα. This observation raises the possibility of a cerebral metabolic phenotype in RTHα and highlights the need for further investigation of CNS metabolic alterations in this disorder.

## 2 Materials and methods

### 2.1 Ethical considerations

This study was approved by the Ethics Committee of the Instituto Nacional de Ciencias Médicas y Nutrición Salvador Zubirán. The research protocol was conducted in accordance with the ethical principles established by the Declaration of Helsinki (30). Written informed consent was obtained from all participants or their legal representatives for genetic testing and for the use and publication of clinical information for scientific research purposes.

### 2.2 Subjects and follow-up studies

Two patients from the same family were included: a 36-year-old woman (proband 1) and her 12-year-old son (proband 2), both evaluated at the same institution. Both presented with clinical features suggestive of altered TH signaling and underwent a comprehensive clinical evaluation, including biochemical studies. Additionally, proband 1 underwent skeletal radiographic evaluation as part of the complementary assessment.

Clinical and biochemical follow-up was performed according to institutional protocols, documenting the clinical course and its correlation with the molecular findings.

### 2.3 Clinical evaluation

A comprehensive clinical evaluation was performed, including a detailed medical history, complete physical examination, and anthropometric measurements. A targeted clinical interview and physical examination were conducted to identify abnormalities involving the neurological, gastrointestinal, hematological, and skeletal systems. Subsequently, clinical findings were systematically integrated into the diagnostic reasoning process.

### 2.4 Biochemical and hormonal analyses

Serum concentrations of TSH, fT4, TT4, TT3, and fT3 were determined using electrochemiluminescence immunoassays. Analyses for proband 1 were performed at the Laboratory of the Instituto Nacional de Ciencias Médicas y Nutrición Salvador Zubirán (Mexico City, Mexico), whereas those for proband 2 were performed at Carpemore International Reference Laboratory (Mexico City, Mexico).

The rT3 was quantified using an enzyme-linked immunosorbent assay (ELISA) at different reference laboratories: Reference Laboratory S.A. (L’Hospitalet de Llobregat, Barcelona, Spain) for proband 1 and Carpemore International Reference Laboratory (Mexico City, Mexico) for proband 2.

Additional biochemical and hormonal studies were performed. In both patients, a complete blood count and lipid profile were obtained. In proband 1, additional assessments included growth hormone, lactate, IGF-1, ACTH, morning serum cortisol (08:00 h), follicle-stimulating hormone (FSH), luteinizing hormone (LH), and testosterone.

Results were interpreted using the reference intervals established by each laboratory, as well as age- and sex-adjusted values according to Wilpert et al. (31) and Yamada et al. (32), particularly for thyroid function tests.

### 2.5 Whole-exome sequencing and sanger sequencing

Genomic DNA was isolated from peripheral blood leukocytes from proband 1 using standard extraction protocols. The obtained DNA was processed using the Whole Exome Solution v2 (SOPHiA GENETICS), according to the manufacturer’s instructions, including DNA fragmentation, adapter ligation, PCR amplification, and enrichment of human coding regions by hybrid capture. Sequencing was performed on the Illumina NextSeq 2000 platform, generating high-quality reads for variant detection in coding regions and adjacent intronic regions. The exome included 19,425 protein-coding genes.

The obtained reads were aligned to the human reference genome GRCh38/hg38 using the WES_v2_hg38 pipeline implemented in the SOPHiA DDM bioinformatics analysis platform. Variant identification, filtering, and annotation were performed using the tools integrated into this platform.

Population frequencies of the identified variants were assessed using public databases, including gnomAD, the 1000 Genomes Project, and ExAC. Clinical databases, including ClinVar and OMIM, were also consulted. As part of the variant prioritization strategy, variants with an allele frequency greater than 1% in reference populations were excluded to focus on rare variants potentially associated with disease.

Variants located in coding regions or canonical splice sites were subsequently prioritized. The potential functional impact of missense variants was assessed using *in silico* prediction tools, including SIFT, PolyPhen-2, MutationTaster, Combined Annotation Dependent Depletion (CADD), and I-Mutant2.0, which estimate the potential effects of amino acid substitutions on protein structure and function. Evolutionary conservation metrics, such as ConSurf, were also considered to assess the conservation of the affected residues across different species.

Final variant prioritization was performed by integrating genetic evidence with genotype–phenotype correlation, considering the clinical features observed in proband 1 using terms derived from the Human Phenotype Ontology (HPO). Candidate variants were classified according to the criteria established by the American College of Medical Genetics and Genomics (33) and described according to the nomenclature recommended by the Human Genome Variation Society (HGVS).

This analysis identified a variant in the *THRA* gene (NM_199334.5:c.662T>C; p.Ile221Thr), with a read depth of 354× and a variant allele fraction of 52.1%, consistent with a heterozygous variant.

Subsequently, the variant identified in proband 1 was evaluated in her 12-year-old son by Sanger sequencing, following standard PCR amplification protocols and analysis of the amplified products by capillary sequencing. Additionally, the variant was queried in the MCPS Variant Browser, which includes data from 9,950 whole genomes and 141,046 exomes from participants in the Mexico City Prospective Study (34).

### 2.6 Assessment of cerebral metabolism using ^18^F-FDG PET/CT

Brain ^18^F-FDG PET/CT was performed using a Discovery 710 PET/CT scanner (GE Healthcare) at the Department of Nuclear Medicine of the Instituto Nacional de Ciencias Médicas y Nutrición Salvador Zubirán (INCMNSZ). After a minimum fasting period of 4 hours, Proband 1’s blood glucose level was measured before the intravenous administration of 370 MBq (10 mCi) of ^18^F-FDG. Proband 1 remained in a quiet, dimly lit room under sensory resting conditions during a 60-minute tracer uptake period, after which PET image acquisition was initiated.

PET data were reconstructed using the ordered subset expectation maximization (OSEM) Vue Point HD (VPHD) algorithm, incorporating point-spread function modeling (SharpIR; 5 mm FWHM) and time-of-flight (TOF) corrections. Reconstruction parameters consisted of 3 iterations, 48 subsets, a 192 × 192 matrix, and a 30-cm field of view (3.3 mm/pixel). A low-dose computed tomography (CT) scan (40 mA; 120 kV) was acquired simultaneously and used exclusively for attenuation correction and anatomical co-registration.

The images were visually evaluated by a Nuclear Medicine specialist with more than 10 years of experience in nuclear neuroscience. Additionally, an age- and sex-matched control subject was included for comparative assessment of cerebral metabolism. The PET/CT study of the control subject was performed using the same scanner and under comparable preparation, acquisition, and reconstruction conditions. Regional cerebral ^18^F-FDG uptake patterns were qualitatively compared between Proband 1 and the control subject, followed by quantitative analysis using manually defined bilateral regions of interest (ROIs) in areas showing visually apparent differences in ^18^F-FDG uptake.

## 3 Results

### 3.1 Clinical characteristics

Proband 1 is a 36-year-old woman and the only child of a mother with a history of hypothyroidism. Pregnancy was uneventful, and she was born at 40 weeks of gestation by vaginal delivery, with Apgar scores of 9 at 1 minute and 9 at 5 minutes. Birth weight was 2,950 g (25th–30th percentile), and birth length was 50 cm (50th percentile). The newborn metabolic screening was normal.

During infancy, she presented with mild psychomotor delay, achieving independent walking at 15 months of age. Before the age of three years, she developed gingival hypertrophy requiring surgical management, at which time anemia was documented. Subsequently, short stature with suboptimal growth progression was observed, prompting thyroid function testing, which yielded results within the normal reference ranges.

During childhood and adolescence, she was evaluated for short stature, anemia, and recurrent episodes of patellar dislocation. The investigations performed, including karyotyping and growth hormone assessment, were normal. In adulthood, she was diagnosed with polycystic ovary syndrome. Other relevant findings included multiple melanocytic nevi, eczema, xerosis, and chronic constipation since childhood.

At the current evaluation, her height was 1.45 m (<3rd percentile) and her weight was 53.8 kg (50th–75th percentile), with a body mass index (BMI) of 25.6 kg/m². Head circumference was 57 cm (90th–97th percentile). A reduced arm span-to-height ratio (0.92) and an increased upper-to-lower segment ratio (1.20) were observed, suggestive of body disproportion, with a relatively long trunk and short extremities. Dysmorphic features included dolichocephaly, temporal and parietal alopecia, a broad forehead, sparse eyebrows, a bulbous nasal tip, a long philtrum, a high-arched palate, and a triangular face. She also had a broad neck and small hands and feet.

Proband 2, the son of proband 1, is a 12-year-old boy. Pregnancy was complicated by intrauterine growth restriction, and he was delivered by cesarean section at 36.5 weeks of gestation, with Apgar scores of 8 at 1 minute and 9 at 5 minutes. The newborn metabolic screening was normal.

During the neonatal period, he presented with poor sucking and subsequently exhibited delayed acquisition of developmental milestones. At three years of age, he was diagnosed with mild central hypotonia and normocytic normochromic anemia. He also has a history of chronic constipation since childhood.

At the current clinical evaluation, his height was 1.53 m (25th–50th percentile), weight was 48.7 kg (75th percentile), and head circumference was 60 cm (>97th percentile), with a BMI of 20.8 kg/m².

The arm span-to-height ratio was slightly reduced (0.93), whereas the upper-to-lower segment ratio was within the normal range (1.00). Dysmorphic features included supraorbital hypoplasia with sparse eyebrows, bilateral epicanthal folds, a broad nasal bridge, a bulbous nasal tip, anteverted nares, a high-arched palate, an interdental diastema, and full cheeks.

Both patients presented with a phenotype characterized by neurodevelopmental abnormalities, dysmorphic features, macrocephaly, chronic constipation, and normocytic anemia (**Table 1**).

**Table 1:** Literature review of clinical data in patients with RTHα compared with the proband 1and the proband 2.

Feature			Proportion of persons w/feature	Proband 1	Proband 2
Neurodevelopment		Developmental delay (DD)/Intellectual disability	34/39	+	+
		Neurobehavioral manifestations	12/31	–	–
		Seizures	3/30	–	–
		Macrocephaly	21/30	relative macrocephaly	absolute macrocephaly
Dysmorphic facial features			21/22	+	+
Gastrointestinal		Constipation	25/34	+	+
Musculoskeletal		Short stature	23/35	+	–
		Wormian bones	6/14	–	NV
Dermatological					
	Eczema		3/13	+	–
	Moles		6/13	+	–
	Thick skin		7/11	–	–
Cardiac abnormality (bradycardia, structural heart disease, and pericardial effusion)			9/23	bradycardia	bradycardia
Anemia			13/14	+	+

+, present; −, absent; NV, not evaluated.

1, 4, 19–29.

### 3.2 Biochemical and hormonal findings

Biochemical evaluation performed at 36 years of age in proband 1 and at 12 years of age in proband 2 revealed subtle abnormalities in the thyroid profile. Both patients had TSH levels within the normal reference ranges, decreased rT3 levels, and an elevated fT3/fT4 ratio.

The fT3/fT4 ratio was 0.439 (75th–90th percentile) in proband 1 and 0.588 (97th percentile) in proband 2. In addition, both patients presented with normocytic normochromic anemia, whereas proband 2 had mild neutropenia. Detailed biochemical results are presented in **Table 2**.

### 3.3 Genetic findings and characterization of the *THRA* variant

Whole-exome sequencing in proband 1 identified a heterozygous variant in the *THRA* gene (NM_199334.5:c.662T>C; p.Ile221Thr), located in exon 7 and corresponding to the genomic position chr17:40086792 T>C (GRCh38). This variant is located within the ligand-binding domain (LBD) of the TRα protein, in its C-terminal region.

The region containing the variant showed adequate sequencing coverage, with a read depth of 354× and a variant allele fraction of 52.1%, consistent with heterozygosity. The variant had not been reported in population or clinical databases, including gnomAD, the 1000 Genomes Project, ExAC, and ClinVar, nor in the literature available at the time of analysis. It was also absent from the MCPS Variant Browser. The affected amino acid position showed a high degree of evolutionary conservation, according to phylogenetic conservation analysis (ConSurf score = 8).

*In silico* prediction tools suggested a deleterious functional effect. PolyPhen-2 classified the variant as probably damaging (score = 0.978), SIFT as deleterious, and MutationTaster as disease-causing. Evolutionary conservation analysis using MutationTaster demonstrated that the Ile221 residue is highly conserved across multiple species, including *Homo sapiens*, *Pan troglodytes*, *Macaca mulatta*, *Mus musculus*, *Danio rerio*, and *Xenopus tropicalis*. In addition, the variant had a CADD score of 28.9, consistent with a predicted deleterious effect.

Additionally, I-Mutant2.0 was used to assess the potential impact of the amino acid substitution on protein stability by calculating the change in Gibbs free energy (ΔΔG) between the wild-type and mutant proteins. This analysis yielded a ΔΔG value of −1.23 kcal/mol at pH 7.0 and 25 °C, suggesting moderate protein destabilization that may have functional implications.

In accordance with the recommendations of the *European Thyroid Association* (ETA) for the interpretation of *THRA* variants (2024), sequence alignment performed using Clustal Omega confirmed that the Ile221 residue of TRα1 corresponds to the homologous Ile275 residue of TRβ1 (RefSeq protein NP_001341641.1). This residue is located within the LBD, a functionally relevant region recognized as a hotspot for pathogenic variants in thyroid hormone receptors. In TRβ, pathogenic variants have been described at nearby residues, including p.Thr273Arg, p.Ile276Leu, p.Thr277Ala, and p.Pro278Leu, which are associated with RTHβ (35).

Although the specific functional effect of the *THRA* p.Ile221Thr variant requires confirmation through experimental studies, the evolutionary conservation of the affected residue, its correspondence to a homologous TRβ residue, and the presence of pathogenic variants at adjacent positions provide complementary biological evidence supporting the plausibility of a deleterious functional effect. Based on the ACMG criteria, the variant was classified as likely pathogenic, with evidence supporting the application of PM1, PM2, PP2, PP3, and PP4.

Subsequently, the variant identified in proband 1 was evaluated in her son (proband 2) by Sanger sequencing, confirming the presence of the same variant.

Finally, no other rare variants with potential pathogenic significance were identified in genes previously associated with resistance to thyroid hormone or in other genes involved in thyroid hormone signaling.

### 3.4 Imaging findings using ^18^F-FDG PET/CT

Proband 1 underwent an assessment of cerebral metabolism using **^18^F**-FDG PET, which demonstrated a pattern of symmetric hypermetabolism in the prefrontal cortex, basal ganglia, and lateral temporal cortex (**Figure 1**). Exploratory quantitative analysis of corresponding bilateral regions of interest (ROIs) showed consistently higher SUVmax values in Proband 1 than in the age- and sex-matched healthy control across all evaluated regions. SUVmax values were higher in the prefrontal cortex (15.44 and 14.81 vs. 10.80 and 11.66), basal ganglia (16.65 and 16.77 vs. 12.10 and 12.16), and lateral temporal cortex (13.40 and 12.81 vs. 9.19 and 8.13), corresponding to descriptive differences of 27.0–57.6% relative to the control (**Figure 1**).

**Figure 1:**
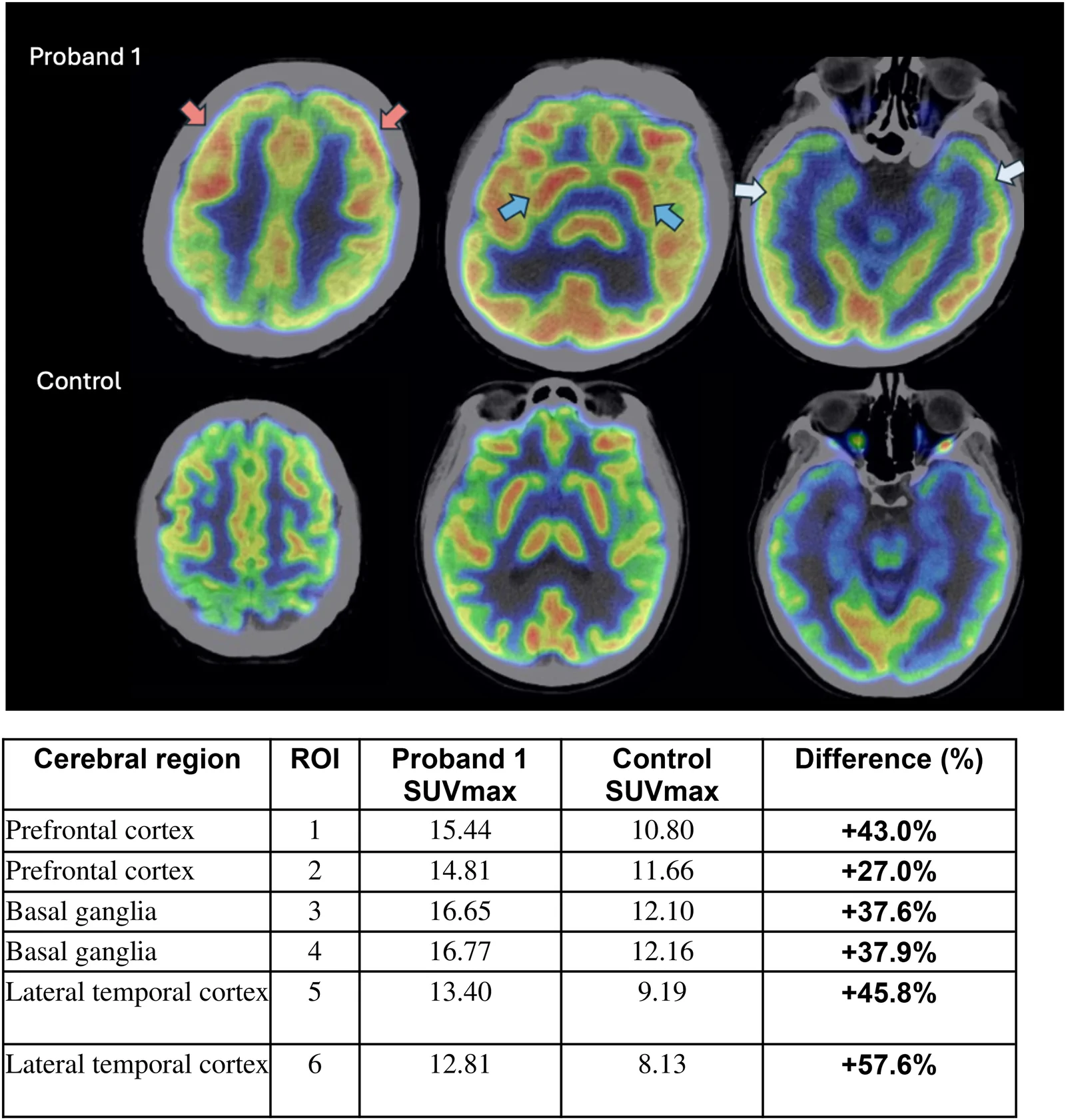
Exploratory cerebral ^18F-FDG PET findings in Proband 1 and an age- and sex-matched control subject. Visual assessment showed symmetrically higher ^18F-FDG uptake in the prefrontal cortex (red arrows), basal ganglia (blue arrows) and lateral temporal cortex (white arrows) in Proband 1 compared with the control subject. Bilateral regions of interest (ROIs) were used for exploratory quantitative assessment of these regions; corresponding SUVmax values and descriptive percentage differences relative to the control are presented in the accompanying table.

The relevance of these regions may be related to the distribution of *THRA* expression in the CNS. Both the *Human Protein Atlas* and the *Hormone Cell Atlas*, resources that enable the characterization of gene expression at the tissue and cellular levels, respectively, show broad *THRA* expression across different regions of the CNS. In particular, the *Human Protein Atlas* reports *THRA* expression levels of up to 238 nTPM in the temporal region of the cerebral cortex and 343.5 nTPM in the basal ganglia. nTPM (normalized transcripts per million) represents a normalized measure of the relative abundance of transcripts within a given tissue. The relatively high expression of *THRA* in these regions raises the possibility of increased susceptibility to alterations in TH-mediated signaling. Given the exploratory nature of the analysis in a single proband and one control subject, the observed hypermetabolic pattern may be related to adaptive changes in cerebral metabolism; however, this possibility warrants further investigation.

## 4 Discussion

In this study, we describe a familial phenotype highly consistent with RTHα, associated with a novel variant in THRA, classified as likely pathogenic according to the criteria established by the ACMG, together with a characteristic biochemical profile and a pattern of cerebral hypermetabolism identified by ^18^F-FDG PET. Since its initial description in 2012, approximately 46 cases of RTHα associated with 25 pathogenic or likely pathogenic variants in the THRA gene have been reported. All of these variants are located within the ligand-binding domain (LBD), in the C-terminal region of the receptor, a region that is critical for its function (**Figure 2**).

**Figure 2:**
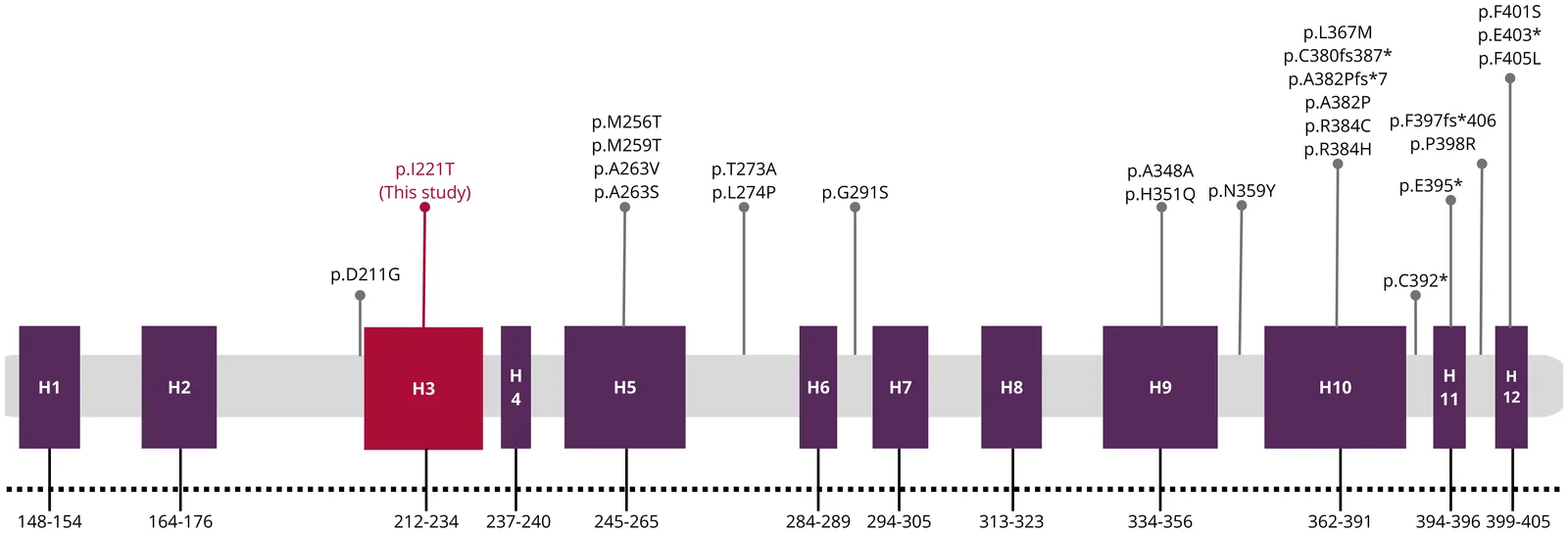
Pathogenic or likely pathogenic variants located within the ligand-binding domain (LBD) of the TRα receptor, a key structural domain involved in TH signaling. This domain is composed of 12 α-helices (H1–H12). The dashed line delineates the extent of the LBD, indicating the length in amino acids of each helix. The distribution of previously reported variants across the domain is shown, along with the variant identified in this study, located in helix H3, a region in which, to the best of our knowledge, no pathogenic variants have been previously reported. Amino acid positions are indicated individually for each helix rather than for the entire LBD. For illustrative purposes, the variants are presented using single-letter amino acid notation, with the variant identified in this study shown as p.I221T.

The variant identified in this study is also located within the LBD of the TRα receptor. This domain consists of 12 α-helices organized into three layers, forming an α-helical sandwich structure that constitutes the hydrophobic ligand-binding pocket (36).

At the C-terminal end of the LBD lies the activation function-2 (AF-2) surface, which is involved in interactions with coregulatory proteins and is formed primarily by helices 3, 4, and 12 (helix 3: residues 212–234; helix 4: 237–240; helix 12: 399–405) (36). Most pathogenic variants reported in this region cluster in helix 12, where functional studies have suggested a dominant-negative mechanism. In these cases, the generation of truncated proteins exposes a hydrophobic cleft that promotes corepressor recruitment, allowing the mutant receptor to bind DNA without adequately activating transcription and thereby interfering with the function of the wild-type receptor. In contrast, missense variants have been associated with impaired coactivator recruitment, ultimately leading to reduced expression of TH-responsive genes (1, 22).

The variant described in this study is located in helix 3; therefore, the specific molecular mechanism through which it contributes to the clinical phenotype remains unclear. However, it is plausible that it alters interactions with coregulatory proteins, potentially in a manner similar to that described for missense variants located in helix 12.

Despite advances in molecular recognition, RTHα remains a diagnostic challenge due to the absence of a pathognomonic biochemical marker. Unlike RTHβ, TRα dysfunction does not significantly alter hypothalamic–pituitary feedback; therefore, circulating TSH concentrations generally remain within the reference range (1, 4, 15, 16, 18, 20, 23, 26, 27).

Although biochemical abnormalities that support the diagnosis may be observed, such as an elevated fT3/fT4 ratio, low rT3 concentrations, and mild hematological abnormalities (normocytic anemia or neutropenia), none of these findings alone is sufficient to establish a diagnosis of RTHα. Consequently, conventional thyroid function tests are often inconclusive, even in the presence of clinical manifestations consistent with tissue-level hypothyroidism (31).

Therefore, recognition relies primarily on identifying a characteristic phenotype, including global developmental delay or intellectual disability, macrocephaly, hypotonia, constipation, dermatological manifestations, and skeletal abnormalities, among other features (37), consistent with the systems predominantly affected in both probands (**Table 1**).

From a biochemical perspective, both probands exhibited subtly altered thyroid profiles, characterized by TSH concentrations close to the 50th percentile, low rT3 levels, and an elevated fT3/fT4 ratio, with values falling at different percentiles in the two individuals (0.439, 75th–90th percentile in the mother; 0.588, 97th percentile in the son) (**Table 2**; **Figures 3A, B**), despite sharing the same heterozygous variant in *THRA* (37).

**Table 2:** Blood analysis of the proband 1 and proband 2 during diagnostic evaluation.

Parameter	Proband 1	Proband 2	Reference range
Thyroid status			
TSH µIU/mL	1.40	2.165	0.19 – 4.92
fT3, pmol/L	5.88	8.55	2.76–6.45
fT4, pmol/L	13.37	14.53	12.9–20.6
fT3/fT4 ratio	0.439	0.588	
Total T3, ng/mL	2.2	1.71	0.58 – 2.0
Total T4, µg/dL	11.0	6.51	5.1– 14.1
Thyroglobulin (Tg), ng/mL	22.89	NV	3.68 – 64.15
rT3, ng/mL	0.16	0.091	0.17 – 0.44
Calcium homeostasis			
Serum calcium, mg/dL	9.5	9.3	8.6 – 9.9
Phosphorus, mg/dL	3.9	NV	2.7 – 4.5
PTH, pg/mL	38.6	NV	12 – 88
Urinary calcium, mg/vol	68	NV	<250
Urinary creatinine, mg/vol	1298	NV	10–15 mg/kg body weight
Vitamin D (1,25-dihydroxy), pg/mL	28.7	NV	19.9 – 79.3
Vitamin D (25-Hydroxy), ng/mL	42	NV	20-50
Hematopoietic evaluation			
Hemoglobin, g/dL	12.0	12.2	13 – 16.6
MCV, fL	93.6	94.4	84 – 99.6
Mean Corpuscular Hemoglobin (MCH), pg	31.8	31	26-34
Leukocytes, 10³/µL	3.5	3.76	3.9 – 10.1
Monocytes, 10³/µL	0.18	0.15	0.3 – 0.9
Neutrophils, 10³/µL	1.82	1.35	2 – 7.2
Peripheral markers			
Cholesterol, mg/dL	202	146.8	<200
LDL, mg/dL	155	91.2	<100
Triglycerides, mg/dL	94	28	<150
Lactate, mmol/L	1.4	NV	0.5 – 1.6
Glucose, mg/dL	78	79	74 – 100
Albumin, g/dL	3.8	4.56	3.5 – 5
Creatine kinase (CPK), U/L	76	NV	29 – 168
Endocrinology			
IGF-1, ng/mL	190.7	NV	81 – 277
Growth hormone, ng/mL	5.25	NV	0.06 – 6.88
ACTH, pg/mL	20	NV	10 – 50
Serum cortisol (08:00 h), µg/dL	23.9	NV	3.7-19.4
Follicle-stimulating hormone (FSH), mIU/L	5.98	NV	Follicular phase: 3.03-8.08
Luteinizing hormone (LH), mIU/mL	6.54	NV	Follicular phase: 1.8-11.78
Testosterone, ng/dL	25.95	NV	9-37

NV, not evaluated.

Both patients exhibited TSH levels around the 50th percentile. However, the fT3/fT4 ratio was 0.439 (75th–90th percentile) in proband 1 and 0.588 (above the 97th percentile) in proband 2. Additionally, both individuals had low rT3 concentrations, while thyroglobulin concentration was within the normal range in proband 1. From a hematological standpoint, normocytic normochromic anemia was identified in both patients, with mild neutropenia observed in proband 2.

**Figure 3:**
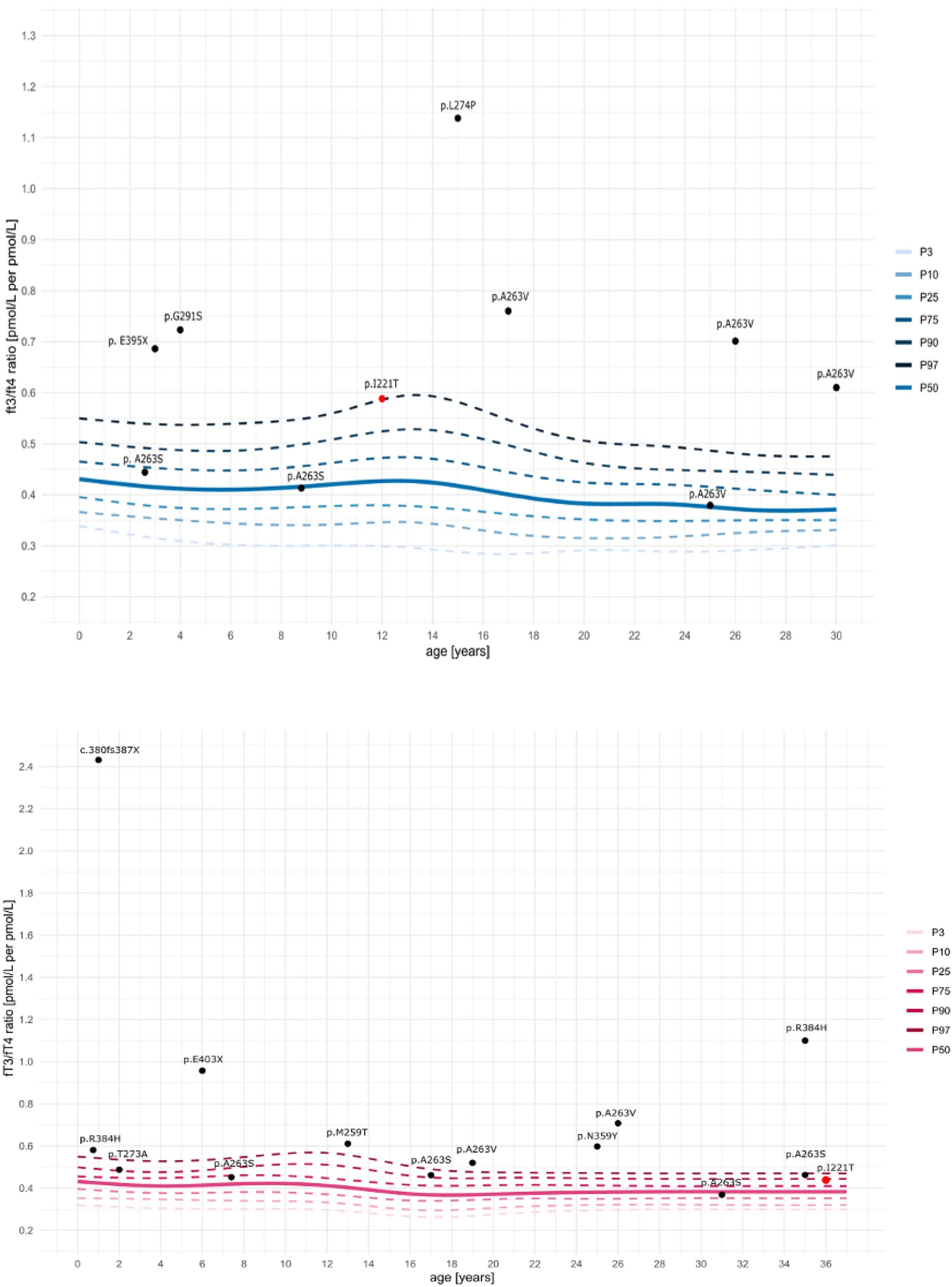
**(A, B)** fT3/fT4 ratio in patients with RTHα with available fT3 and fT4 values reported in the literature. The graphs are based on normal reference values established by Wilpert et al. (31) and Yamada et al. (32). The values corresponding to proband 1 (36 years) and proband 2 (12 years) are shown within their respective age groups and highlighted as red dots. For illustrative purposes, variants were represented using single-letter amino acid notation. **(A)** Male patients, fT3/fT4 ratio as a function of age. **(B)** Female patients, fT3/fT4 ratio as a function of age.

This observation prompted a review of published RTHα cases for which complete thyroid profiles were available. In this analysis, 60% of males and 53% of females had an fT3/fT4 ratio above the 97th percentile for age (**Figures 3A, B**). However, a substantial percentage of patients did not show this increase, highlighting the need to identify additional biochemical markers or complementary tools to optimize the diagnostic approach (1, 15–18, 20, 23, 25–27).

Importantly, this biochemical profile is not specific to RTHα; therefore, diagnosis requires the integration of clinical, biochemical, and molecular findings. Moderate-to-severe iodine deficiency represents an important differential diagnosis, as it may be associated with elevated TSH, normal or decreased fT4, elevated fT3, and low rT3, thereby mimicking the biochemical profile observed in RTHα. However, the presence of goiter, markedly elevated serum thyroglobulin concentrations, and decreased urinary iodine levels may help distinguish iodine deficiency from RTHα (38–40). Another relevant differential diagnosis is primary congenital hypothyroidism due to defects in thyroid hormonogenesis, caused by pathogenic variants in genes such as *DUOX1, DUOX2, DUOXA1, DUOXA2, SLC5A5, SLC26A4, SLC26A7, TPO*, and *IYD*. Although these disorders are classically characterized by elevated TSH and decreased fT4, mild or partial forms may exhibit a biochemical profile resembling that of RTHα. In such cases, the presence of goiter and elevated serum thyroglobulin concentrations may favor a diagnosis of a thyroid hormonogenesis defect (40–45).

By contrast, central hypothyroidism may present with clinical manifestations similar to those of primary hypothyroidism, although goiter is uncommon. Biochemically, it is characterized by low fT4 concentrations with inappropriately low, normal, or only mildly elevated TSH levels, whereas fT3 may be low or within the normal range. In contrast, patients with RTHα exhibit distinctive clinical features, together with fT3 and TT3 concentrations at the upper end of the reference range, which may help differentiate RTHα from central hypothyroidism (46).

Finally, Allan–Herndon–Dudley syndrome (OMIM #300523), associated with pathogenic or likely pathogenic variants in *SLC16A2*, located at Xq13.2, is characterized by elevated fT3 concentrations, decreased rT3 levels, and an fT3/fT4 ratio above the 97th percentile, thereby sharing a biochemical profile similar to that of RTHα. However, it can be distinguished by the absence of peripheral manifestations of hypothyroidism. Although both disorders predominantly affect the CNS, the severity and clinical spectrum depend on the specific function of the affected gene (31, 47).

The potential cerebral impact of RTHα is of particular interest, given that peripheral hormone parameters do not necessarily directly reflect TH action at the level of the CNS. As previously reported, both hypothyroidism and hyperthyroidism can reduce cerebral glucose uptake, a phenomenon detectable by ^18^F-FDG PET, and can lead to metabolic alterations independent of peripheral blood glucose levels (48–50). To determine whether subtle alterations in a euthyroid state were associated with a specific cerebral metabolic pattern, ^18^F-FDG PET was performed in proband 1. This study revealed a pattern of hypermetabolism in the prefrontal cortex, basal ganglia, and lateral temporal cortex, which may reflect regional differences in compensatory mechanisms in response to altered TH signaling (**Figure 1**). This finding should be interpreted as an individual observation rather than as an established cerebral metabolic feature of RTHα. Given that only one proband and one control subject were evaluated and no longitudinal assessment was available, it is not possible to determine whether this pattern is directly attributable to altered TRα-mediated signaling.

However, experimental models provide a relevant biological basis for considering potential alterations in cerebral metabolism associated with impaired TRα signaling. Itoh and colleagues demonstrated that young mice heterozygous for the dominant-negative TRαPV mutation (TRαPV/+) exhibit a 57–72% reduction in regional cerebral glucose utilization, particularly in the cortex and caudate-putamen, whereas TRβPV/PV mice showed no significant alterations compared with controls (51). In our patient, by contrast, ^18^F-FDG PET showed hypermetabolism in the prefrontal cortex, basal ganglia, and lateral temporal cortex, with no hypometabolic regions identified (**Figure 1**). This difference should be interpreted in the context that the TRαPV model represents severe functional impairment of TRα during early development, whereas our patient carries a heterozygous *THRA* missense variant identified in adulthood, which may allow the involvement of long-term adaptive mechanisms.

RTHα resulting from TRα1 dysfunction should not necessarily be regarded as equivalent to global cerebral hypothyroidism. The specific impairment of this receptor affects only a subset of T3-regulated gene programs and may result in selective transcriptional responses, in contrast to systemic hypothyroidism, which alters a broader spectrum of genes (52). In addition, cerebral TH signaling depends on the relative expression of TR, intracellular T3 availability, and local deiodinase activity. Although TRα1 is the primary mediator of T3 action in the brain, TRβ is expressed in several brain regions and may regulate gene sets that partially overlap with those regulated by TRα1. This capacity, together with its regional expression patterns, may contribute to partially preserving T3-dependent signaling in the setting of a heterozygous *THRA* alteration, without fully compensating for TRα1 dysfunction (52).

The hypermetabolic regions identified in our patient overlap with brain regions showing relatively higher *DIO2* and lower *DIO3* expression in human brain transcriptomic datasets from the Human Protein Atlas (HPA). Because DIO2 promotes the intracellular conversion of T4 to T3, whereas DIO3 limits TH availability, regional differences in deiodinase expression may influence local T3 availability and, consequently, modulate TH signaling through the remaining functional receptors. Notably, *Dio3* has been shown to be a T3-regulated gene in a TRα1-dependent manner in the embryonic mouse cerebral cortex, supporting the existence of local mechanisms that fine-tune TH signaling (53, 54). Additionally, the actions of THs are not limited to their direct effects mediated by TR in target tissues but also include central actions through the hypothalamus, which coordinates the peripheral metabolic response via autonomic pathways, including the sympathetic nervous system. Both pathways are integrated into the regulation of energy expenditure and metabolic homeostasis (55). Other mechanisms, such as changes in neuronal activity and glucose transport, may contribute to the maintenance or enhancement of regional glucose metabolism (50, 56, 57). Collectively, these mechanisms provide a biological basis for considering the regional hypermetabolism observed in our patient and may contribute to explaining the absence of a generalized hypometabolic pattern.

Although pathogenic or likely pathogenic variants in *THRA* and those in *SLCO1C1* (OMIM #613389) can both disrupt TH signaling in the CNS, they differ in the level at which the defect occurs. *SLCO1C1* defects limit TH transport and, consequently, intracerebral TH availability, whereas alterations in *THRA* primarily affect the cellular response to T3. This difference may promote greater regional heterogeneity in TH signaling in RTHα, particularly through local T3 availability and the involvement of compensatory mechanisms, and may therefore contribute to distinct regional metabolic patterns, including the hypermetabolism observed in our proband, in contrast to the predominantly hypometabolic patterns described in transport defects (58).

Taken together, these mechanisms may help explain the metabolic pattern observed in our proband, although their pathophysiological relevance cannot yet be established. Further ^18^F-FDG PET studies in patients with RTHα are needed to determine whether this pattern represents a reproducible feature of the disorder (50, 56, 57).

Beyond its effects on the CNS, the role of TH signaling in skeletal development is also well established. TRα dysfunction or absence is associated with growth retardation, delayed bone age, impaired mineralization, and decreased bone mineral density, confirming the central role of TH in chondrogenesis and proper skeletal development. Murine models have demonstrated that, even in the presence of normal systemic thyroid status, impaired TRα-mediated signaling leads to a state of “skeletal hypothyroidism.” During growth, T3 exerts predominantly anabolic effects on chondrocytes and osteoblasts; in adulthood, in contrast, its action becomes predominantly catabolic, promoting bone resorption and remodeling (59, 60).

In mice lacking TRα, delayed ossification and reduced mineralization are observed during early developmental stages; subsequently, during adulthood, an osteosclerotic phenotype develops, characterized by thickened trabeculae, persistence of calcified cartilage, reduced osteoclast number and activity, and, consequently, denser bones with impaired remodeling. These findings are consistent with the manifestations described in patients with RTHα—including cranial hyperostosis, cortical thickening of shortened long bones, and frontal bossing (15–17, 20, 23)—as well as with the features observed in proband 1, who presented with cortical thickening of the long bones and cranial hyperostosis (**Figure 4**).

**Figure 4:**
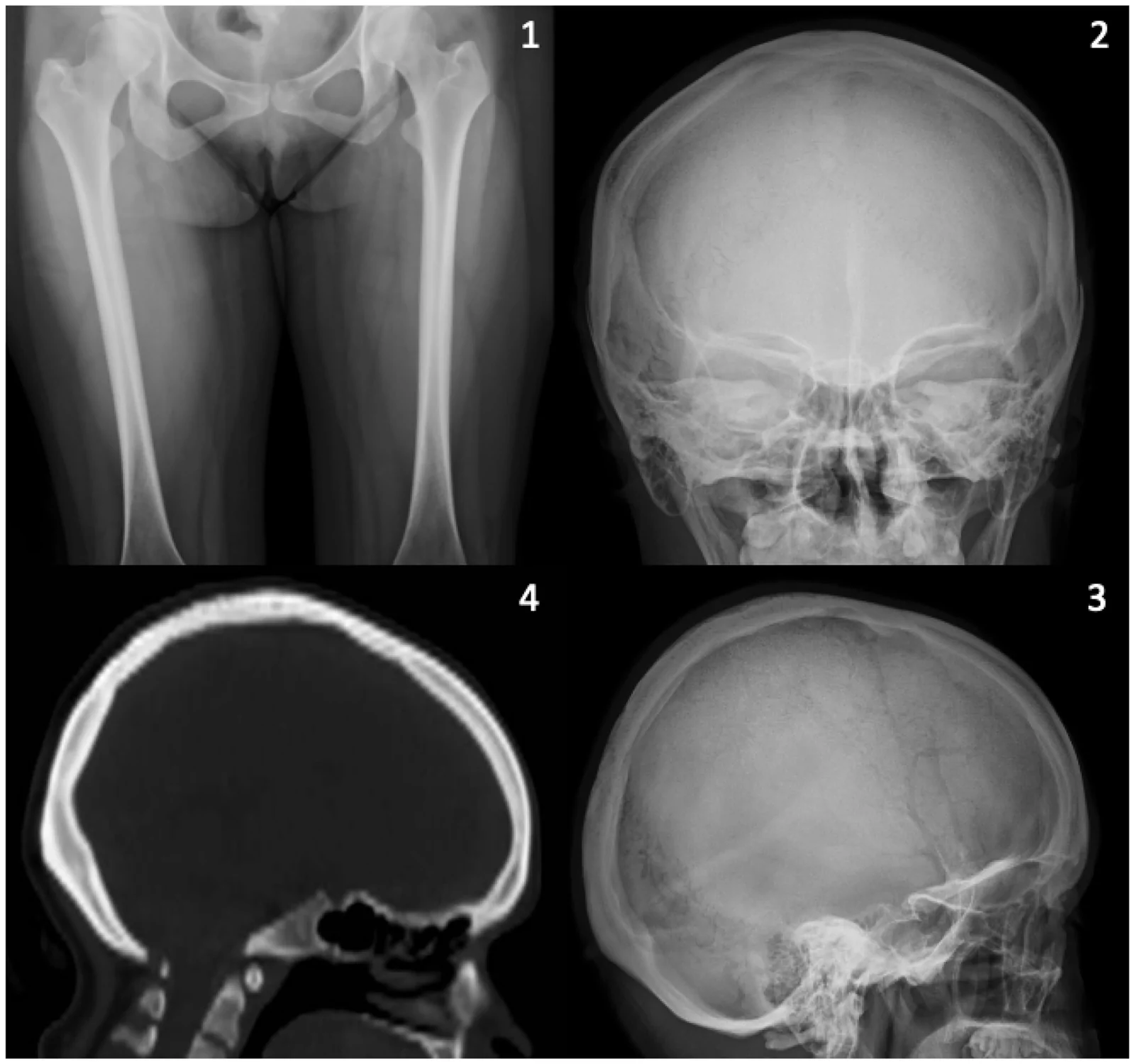
Skeletal survey of proband 1. Anteroposterior (AP) radiograph (1) of both femurs showing cortical thickening of the diaphyses; skull radiographs in AP (2) and lateral (3) projections, and sagittal cranial computed tomography (4), demonstrating calvarial thickening (cranial hyperostosis).

### 4.1 Study limitations

This study has the following limitations. Brain metabolic evaluation using ^18^F-FDG PET was performed in a single patient, precluding generalization of the findings. In addition, no post-treatment PET assessment was available, limiting the evaluation of whether the observed metabolic pattern changes following TH replacement. Although the *THRA* variant was classified as likely pathogenic and segregated with the familial phenotype, no *in vitro* functional studies were conducted to assess its impact on receptor activity. In addition, the small cohort size, limited to a single family, restricts the exploration of phenotypic variability. Future studies including a larger number of patients and functional analyses will be necessary to confirm the proposed mechanisms and the clinical relevance of the observed metabolic findings.

## 5 Conclusion

Overall, RTHα is a rare and likely underrecognized disorder characterized by substantial clinical and biochemical heterogeneity. Because no single pathognomonic biochemical marker exists, diagnosis requires careful integration of the clinical phenotype with subtle alterations in TH parameters. Recognition is particularly important in individuals presenting with suggestive neurological, developmental, gastrointestinal, or skeletal features despite apparently reassuring routine thyroid function tests.

Available experimental and clinical evidence suggests that early identification of RTHα may have meaningful therapeutic implications. Previous clinical reports suggest that timely TH treatment may improve selected clinical manifestations, particularly neurodevelopmental function, motor features, growth, and aspects of skeletal health.

Taken together, these observations highlight the importance of early diagnosis, molecular confirmation when appropriate, and individualized long-term multidisciplinary follow-up in patients with RTHα.

## Data Availability

The raw data supporting the conclusions of this article will be made available by the authors, without undue reservation.
